# Validation and Development of a New Automatic Algorithm for Time-Resolved Segmentation of the Left Ventricle in Magnetic Resonance Imaging

**DOI:** 10.1155/2015/970357

**Published:** 2015-06-21

**Authors:** Jane Tufvesson, Erik Hedström, Katarina Steding-Ehrenborg, Marcus Carlsson, Håkan Arheden, Einar Heiberg

**Affiliations:** ^1^Department of Clinical Physiology, Lund University Hospital, Lund University, 221 85 Lund, Sweden; ^2^Department of Numerical Analysis, Centre for Mathematical Sciences, Faculty of Engineering, Lund University, 221 00 Lund, Sweden; ^3^Department of Diagnostic Radiology, Lund University Hospital, Lund University, 221 85 Lund, Sweden; ^4^Department of Biomedical Engineering, Faculty of Engineering, Lund University, 221 00 Lund, Sweden

## Abstract

*Introduction.* Manual delineation of the left ventricle is clinical standard for quantification of cardiovascular magnetic resonance images despite being time consuming and observer dependent. Previous automatic methods generally do not account for one major contributor to stroke volume, the long-axis motion. Therefore, the aim of this study was to develop and validate an automatic algorithm for time-resolved segmentation covering the whole left ventricle, including basal slices affected by long-axis motion.* Methods.* Ninety subjects imaged with a cine balanced steady state free precession sequence were included in the study (training set *n* = 40, test set *n* = 50). Manual delineation was reference standard and second observer analysis was performed in a subset (*n* = 25). The automatic algorithm uses deformable model with expectation-maximization, followed by automatic removal of papillary muscles and detection of the outflow tract. *Results.* The mean differences between automatic segmentation and manual delineation were EDV −11 mL, ESV 1 mL, EF −3%, and LVM 4 g in the test set. *Conclusions.* The automatic LV segmentation algorithm reached accuracy comparable to interobserver for manual delineation, thereby bringing automatic segmentation one step closer to clinical routine. The algorithm and all images with manual delineations are available for benchmarking.

## 1. Introduction

Cardiovascular magnetic resonance (CMR) imaging can provide diagnostic information about the left ventricle (LV) with clinical parameters such as end-diastolic volume (EDV), end-systolic volume (ESV), ejection fraction (EF), left ventricular mass (LVM), stroke volume (SV), cardiac output (CO), peak ejection rate, peak filling rate, and regional wall thickening. To extract these clinical parameters current clinical practice is to perform endocardial and epicardial delineations manually, which is time consuming and therefore often only performed in end-diastole and end-systole [1]. However, delineations in two frames only will not give peak filling rate and peak ejection rate which require time-resolved segmentation. There is also a need for segmentation throughout the cardiac cycle in the evaluation of patients with dyssynchrony, for example, to determine first and last segments with contraction [2]. With a typical time resolution of 30 frames per heartbeat, time-resolved manual delineation thus requires 15 times longer than manual delineation in only end-diastole and endsystole.

Automatic segmentation is desirable to reduce both analysis time and observer dependency. The continued need for manual delineation indicates that previously suggested automatic methods do not give satisfactory results. Often they do not cover the whole LV and there is a need for much manual interaction. Petitjean and Dacher [3] pointed out that it is hard to conclude on superiority of any of the previously proposed methods since the results are obtained on images with different quality and in different patient populations. Also, the methods are validated using different error measurements, both clinical parameters and image processing error measurements. In midventricular slices the errors were by Petitjean and Dacher concluded to be generally satisfactory [3]. However, basal and apical slices generally yield higher errors [4].

Inclusion of all basal slices in the segmentation is important since the atrioventricular plane displacement is a major contributor to cardiac pumping [5, 6]. The long-axis motion causes the outflow tract to move in and out of the imaging plane during a cardiac cycle. Thereby, segmentation of endocardial and epicardial borders become more difficult in the most basal slices and an automatic detection of the long-axis motion is needed. To our knowledge three studies have included slices with outflow tract [4, 7, 8]. However, in the study by Jolly et al. [4] the detection of outflow tract was not defined, in the study by Hu et al. [7] the outflow tract was detected but the detection of long-axis motion was not defined, and finally in the study by Codella et al. [8] the user defined the most basal slice in both end-diastole and end-systole and thus the long-axis motion was not detected by the algorithm.

The aims of this study were (1) to develop an algorithm for time-resolved LV segmentation covering the whole LV, from the basal slices with outflow tract to the apex, and (2) to validate this new algorithm with regard to clinical parameters and image processing errors for comparison to previous algorithms, and (3) to provide software as well as images with manual delineation to enable benchmarking for future algorithms.

## 2. Methods

### 2.1. Study Population and Design

In total 90 subjects were included in the study, both patients referred for clinical evaluation of known or suspected coronary artery disease as well as healthy subjects and athletes. The subjects were scanned using a 1.5T MR scanner (Philips Intera CV, Philips, Best, The Netherlands) with a cardiac synergy coil. The sequence used was a balanced steady state free precession (bSSFP) sequence with retrospective ECG triggering. Typical imaging parameters were repetition time 2.8 ms, echo time 1.4 ms, flip angle 60°, SENSE factor of 2, spatial reconstructed resolution of 1.4 × 1.4 × 8 mm, and 30 reconstructed time frames per cardiac cycle (acquired spatial resolution 2.3 × 2.7 × 8 mm and temporal resolution 50 ms).

The subjects were divided into a training set (*n* = 40) and a test set (*n* = 50). The training set was used for the development and optimization of the algorithm, and the test set was used to validate the algorithm. The training set consists of 20 patients, 13 healthy volunteers, and 7 athletes. The test set consists of 20 patients, 20 healthy volunteers, and 10 athletes. Manual segmentation was performed for all slices in end-diastole and end-systole in both the training set and the test set by an experienced clinician (ErH with 14 years of CMR experience). The training set was reviewed for consensus by another experienced clinician (HA with 20 years of CMR experience). A subset of 25 subjects from the test set (10 patients, 10 healthy volunteers, and 5 athletes) was used for second observer analysis, by another experienced clinician (MC with 14 years of CMR experience).

Image quality was representative of images likely found in daily clinical routine. Differences in clinical left ventricular parameters EDV, ESV, EF, LVM, SV, and CO between patients and healthy subjects in the test set were nonsignificant for all parameters except SV. The training set and test set with manual delineations are available upon request to the corresponding author to enable direct comparison to other methods.

### 2.2. Automatic Segmentation Algorithm

An automatic algorithm was developed for time-resolved segmentation of the endocardial and epicardial borders of the LV covering all ventricular slices from the most basal slices with outflow tract to the apex. The user input required by the algorithm is the definition of slices to analyze as the most basal slice and most apical slice containing any myocardium. The slices to analyze were in this study automatically selected to be the same as selected for the reference manual delineation. The algorithm was implemented in the freely available cardiac image analysis software Segment (http://segment.heiberg.se/) [9].

The algorithm is based on a deformable model framework. Deformable model is a segmentation method based on the idea of deforming a model to the location and shape of minimal energy in a force field. The model to deform is in this study a model of either the endocardial or the epicardial border. The initialization of the model is based on the image to segment and the initialization is further described in Step 3  of the algorithm. The force field which deforms the model consists of a weighted sum of image-dependent and model-dependent forces. The image-dependent forces are a balloon force image, calculated from image intensities and an edge force image, based on edge detection. The model-dependent forces are based on the curvature within the slice, between adjacent slices and between time frames of the cardiac cycle. The weighting of the forces in the deformable model was optimized based on the training set to obtain parameters suitable for the image type and quality in the training set. The optimization is further described in Deformable Model Optimization section.

Step 1 of the automatic segmentation algorithm is to define the center of the left ventricle, which is needed to calculate the balloon images in Step 2 and to initialize the deformable model in Step 3. Steps 4 and 5 use the deformable model for endocardial and epicardial segmentation. In Steps 6–8 the segmentation resulting from the deformable model is modified to account for the papillaries and the outflow tract. All steps are further described below.

The steps of the algorithm are as follows:

1. definition of the left ventricular center point,
2. calculation of balloon image,
3. initialization of segmentation,
4. endocardial segmentation,
5. epicardial segmentation,
6. exclusion of detached papillaries,
7. detection of outflow tract,
8. exclusion of attached papillaries.

*Definition of the Left Ventricular Center Point (Step 1).* First the center of the whole heart is defined from the largest bright region by smoothing and thresholding the image. The center of the left ventricular cavity is then defined as the center of gravity of the large, bright region closest to the right of the whole heart center point.

**Figure 1:**
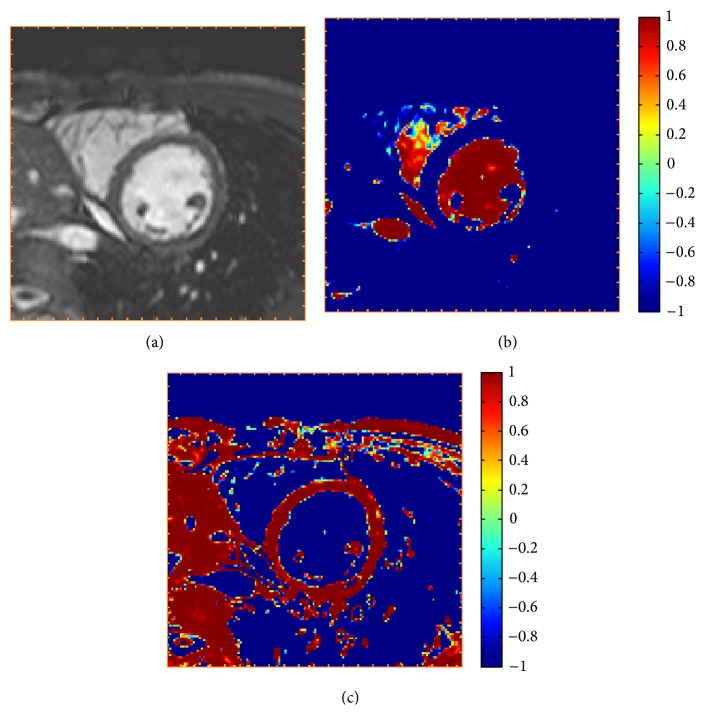
Calculation of balloon force (Step 2). A midventricular slice of a short-axis stack (a) and the endocardial (b) and epicardial (c) balloon force images calculated in Step 2 with the automatic algorithm. The color scale indicates how the deformable model should expand to include pixels with positive values (red) and contract to exclude pixels with negative values (blue).

*Calculation of Balloon Image (Step 2)*. The balloon force, which is the most important part of the deformable model, is defined using an expectation maximization (EM) algorithm. The balloon force drives the expansion and contraction of the curve and thereby should be a distinction between what to include and exclude in the endocardial and epicardial segmentations. The balloon image is mapped from the image intensities by estimating the distribution of intensities in the images. For endocardial segmentation the intensity distributions for blood and myocardium are estimated. In addition for epicardial segmentation, the intensity distribution of tissues surrounding the left ventricle is estimated. An EM-algorithm was utilized to estimate assumed Gaussian distribution of intensities for blood, myocardium, and surrounding tissues. As an initialization to the EM-algorithm, the mean and standard deviation for the intensity of blood were estimated in a cylinder with radius of 10 mm placed at the left ventricular center point. The endocardial balloon image was calculated as the Gaussian distribution for blood divided by the sum of the Gaussian distributions for blood and myocardium. The epicardial balloon image was calculated as the Gaussian distribution for myocardium divided by the sum of the Gaussian distributions for blood, myocardium, and surrounding tissues. The balloon force is positive for intensity values to include and negative for intensities to exclude and the balloon force was rescaled to the interval −1 to 1.  Figure 1 shows the results from calculation of the balloon image.

*Initialization of Segmentation (Step 3). *To initialize both the endocardial and epicardial segmentations the endocardial balloon image is used. The endocardium is initialized at an estimated midmural center line and the epicardium is initialized as an estimate of the epicardial border. The initialization is divided into five substeps.

Thresholding the endocardial balloon image at zero to find regions representative of blood. Balloon force zero is representative of the probability of myocardium being equal to the probability of blood.
Finding the left ventricular blood pool as a region in the thresholded image which surrounds the left ventricular center point.
Estimating the endocardial border as the convex hull of the left ventricular blood pool. The convex hull is an estimation of the endocardial border excluding papillaries.
Estimating the left ventricular wall thickness in each time frame by finding the mean distance from the initial curve to the right ventricular blood pool.
(a) Expanding the endocardial border estimated in Step 3 by a half wall thickness to get the endocardial initialization.
(b) Expanding the endocardial border estimated in Step 3 by one full wall thickness to get the epicardial initialization.

**Figure 2:**
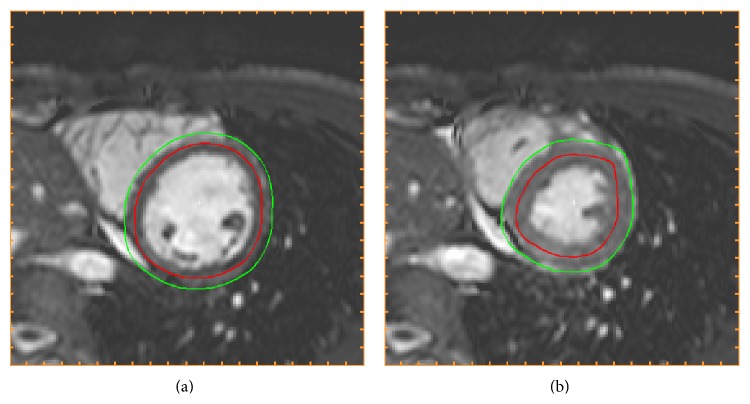
Initialization of segmentation (Step 3). The initializations of endocardial (red) and epicardial (green) borders resulting from Step 3 in the algorithm, shown in end-diastole (a) and end-systole (b) in the midventricular slice also used for Figure 1. The endocardial initialization is an estimation of the midmural line and the epicardial initialization is an estimation of the epicardial border.

Figure 2 shows the initialization of endocardium and epicardium.

*Endocardial Segmentation (Step 4). *For endocardial segmentation, the deformable model is used with endocardial initialization, endocardial balloon force, and weighting of the forces optimized for endocardial segmentation. The deformable model formalism used has previously been described [10]. In short, in the deformable model, the node forces are normalized and projected onto the curve normal and the parameterization of the node points is kept equidistant. The deformable model includes balloon force, edge force, curvature force, temporal acceleration, and damping forces.

*Epicardial Segmentation (Step 5). *For epicardial segmentation, the deformable model is used with epicardial initialization, epicardial balloon force, and weighting of the forces optimized for epicardial segmentation. The epicardial balloon force is negative for blood and other tissues surrounding the myocardium and hence the deformable model will contract to not include any blood. To get an epicardial segmentation which expands outwards from the endocardial segmentation the epicardial balloon force was modified to be zero for all pixels inside the endocardium.

*Exclusion of Detached Papillaries (Step 6).* For measurement of ventricular volumes, the clinical standard is to exclude the papillaries from the myocardium and therefore the algorithm should also exclude the papillaries. Since papillary muscles have the same intensity as myocardium and the main driving force in the deformable model, the balloon force, is based on intensity, the algorithm may have difficulties with excluding the papillaries from the myocardium hence including the papillaries within the endocardial segmentation. The exclusion of papillaries is divided into two steps, this step and Step 8. In this step, detached papillaries are included inside the endocardial segmentation by taking the convex hull of the endocardial segmentation and refining the segmentation. The segmentation is refined by using the deformable model with a modified endocardial balloon force. The endocardial balloon force is modified by setting the balloon force to zero for papillaries, which are detected as pixels inside the convex hull with a negative balloon force.

**Figure 3:**
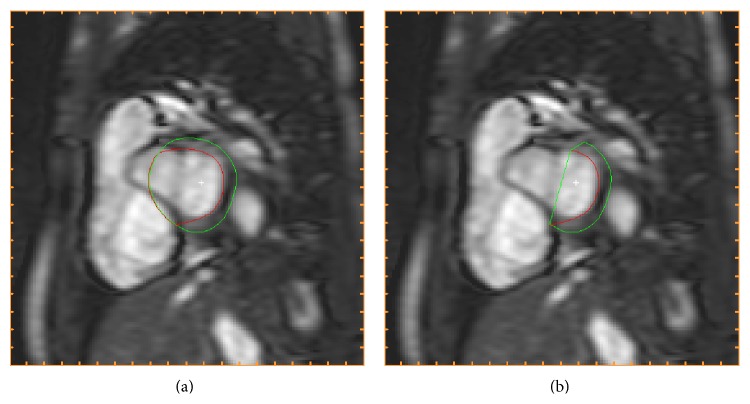
Detection of outflow tract (Step 7). The endocardial (red) and epicardial (green) segmentations are shown prior to the detection of outflow tract in Step 7 (a) and after adjustment of segmentation for presence of outflow tract (b) in the same basal slice in end-diastole.

*Detection of Outflow Tract (Step 7). *The deformable model gives endocardial and epicardial segmentation in all selected slices and time frames. Thereafter, long-axis motion and outflow tract are detected and in the basal slices the segmentation is adjusted accordingly. The detection of the long-axis motion is based on detecting sectors in the basal slices for which the intensities between the endocardial and epicardial segmentation are not typical for myocardium and sectors with a mean wall thickness of less than 2 millimeters. Basal slices were for detection of outflow tract defined as the most basal 40% of the ventricular length in end-diastole and all slices were divided into 24 sectors circumferentially. The intensities in basal slices are compared to intensities in all slices. Sectors with a mean intensity 2 SD above the mean are marked as sectors to remove. Sectors can only be marked as sectors to remove if the sectors are also removed in a more basal slice. Sectors to be removed are smoothed over time and circumferentially in each slice and a morphological opening is performed to get a cohesive region to remove. To remove the marked sectors a straight line is drawn for both endocardium and epicardium. Thereby a D-shaped segmentation is obtained after adjustment for presence of outflow tract.  Figure 3 shows the segmentation in a basal slice before and after the detection of outflow tract.

*Exclusion of Attached Papillaries (Step 8). *To exclude papillaries which are closely attached to the left ventricular wall in the segmentation, it is not sufficient to take convex hull and refine as in Step 6 since there is no blood volume which can guide the deformable model on where to expand the segmentation. Therefore, in this step an expansion of the endocardial segmentation is calculated based on a constant papillary volume over time and a similar position of the papillary muscles over time. Sectors with a lower papillary volume inside the endocardial segmentation than in end-diastole are expanded to include more papillary volume. The long-axis displacement found when detecting the outflow tract in Step 7 is used to map slices in end-diastole to the corresponding slice in all other timeframes. Expansion of the endocardial segmentation is restricted to slices below the outflow tract in order to not falsely take the mitral valve into account as papillary muscle.

### 2.3. Deformable Model Optimization

Weighting of the forces in the deformable model was optimized with a steepest-descent method in a 2-factorial design by using the images in the training set with manual delineation as reference standard. For the endocardial segmentation the error to minimize was the sum of the relative errors of the end-diastolic volume, and the relative number of falsely segmented pixels in end-diastole by comparing the automatic segmentation to manual delineation. Only the end-diastolic errors were included since the errors in end-systole are largely influenced by the presence of papillary muscles which is not especially accounted for within the deformable model.

For the epicardial segmentation the error to minimize in the optimization was the sum of the relative errors of left ventricular mass, in end-diastole and end-systole, and the relative number of falsely segmented pixels, in end-diastole and end-systole. In order to not take into account any volumetric errors in left ventricular mass given by the automatic endocardial segmentation, the left ventricular mass was during optimization calculated using the manual delineation of endocardium.

### 2.4. Statistical Analyses

In the test set the difference between manual delineation and automatic segmentation was computed for the clinical parameters EDV, ESV, EF, LVM, SV, and CO as well as the image processing error measurements dice similarity coefficient (DSC) [11] and point to curve distance (P2C).

The errors for clinical parameters are given both as absolute errors and as percentage of the result from the manual delineation. Paired *t*-test was performed with significance level *P* < 0.05 to test for difference compared to manual delineations. A linear regression was performed for the clinical parameters and a regression *R*-value and corresponding *P*-value were calculated. The DSC is calculated as two times the volume of the intersection of two regions divided by the sum of the volume for those regions [11]. The DSC is therefore 0 if the regions do not overlap and 1 if the regions overlap perfectly. The P2C error was calculated as the distance between two borders in each slice and time frame where both borders were present. To calculate the distance both borders were resampled to be represented by 80 points spaced at every 4.5 degrees. The DSC and P2C errors were calculated between automatic segmentation and manual delineation for both endocardial and epicardial segmentation separately. The DSC and P2C error were calculated as a mean over all slices in both end-diastole and end-systole as well as separately for end-diastole and end-systole and separately for basal, midventricular, and apical slices. Basal, midventricular, and apical slices were defined as one third each of the ventricular length in both end-diastole and end-systole. All errors were reported as mean ± SD.

In the subset for which second observer manual delineation was performed the same error calculations as for the full test set were performed for automatic segmentation versus reference manual delineation and for second observer manual delineation versus reference manual delineation.

## 3. Results

**Figure 4:**
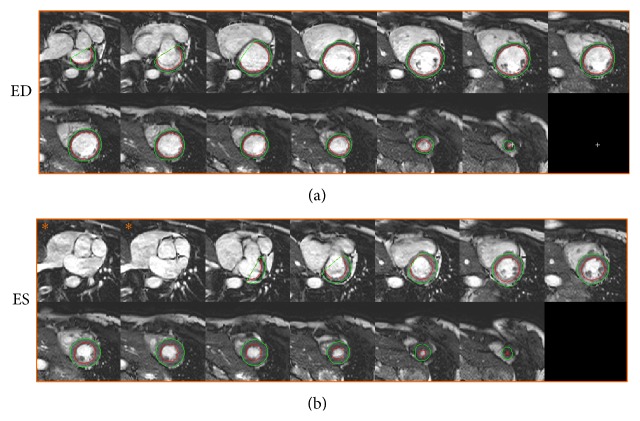
Example of segmentation in end-diastole and end-systole. An example of automatic segmentation is shown in end-diastole (a) and end-systole (b). Each panel shows the short axis stack covering the left ventricle from base to apex with endocardial (red) and epicardial (green) segmentations. Note how the outflow tract has moved out of the two most basal slices in end-systole (b, images marked *∗*), compared to end-diastole (a) and that the algorithm has automatically corrected for this long-axis motion.

**Figure 5:**
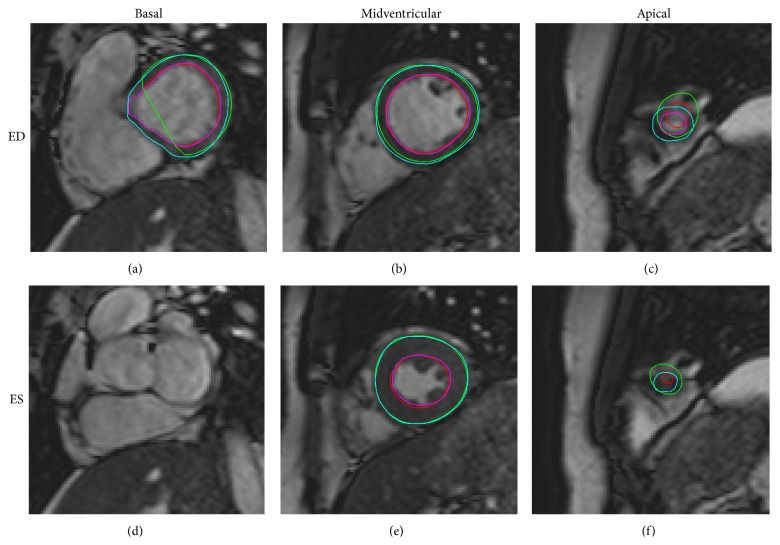
Automatic segmentation compared to manual delineation in a basal, midventricular and apical slice. Automatic segmentation (endocardium in red and epicardium in green) and manual delineation (endocardium in pink and epicardium in light blue) shown in end-diastole ((a), (b), and (c)) and end-systole ((d) (e), and (f)) for the most basal slice with outflow tract moving out of the imaging plane ((a), (d)), a midventricular slice with papillaries ((b), (e)) and an apical slice with minimal lumen in end-systole ((c), (f)).

**Figure 6:**
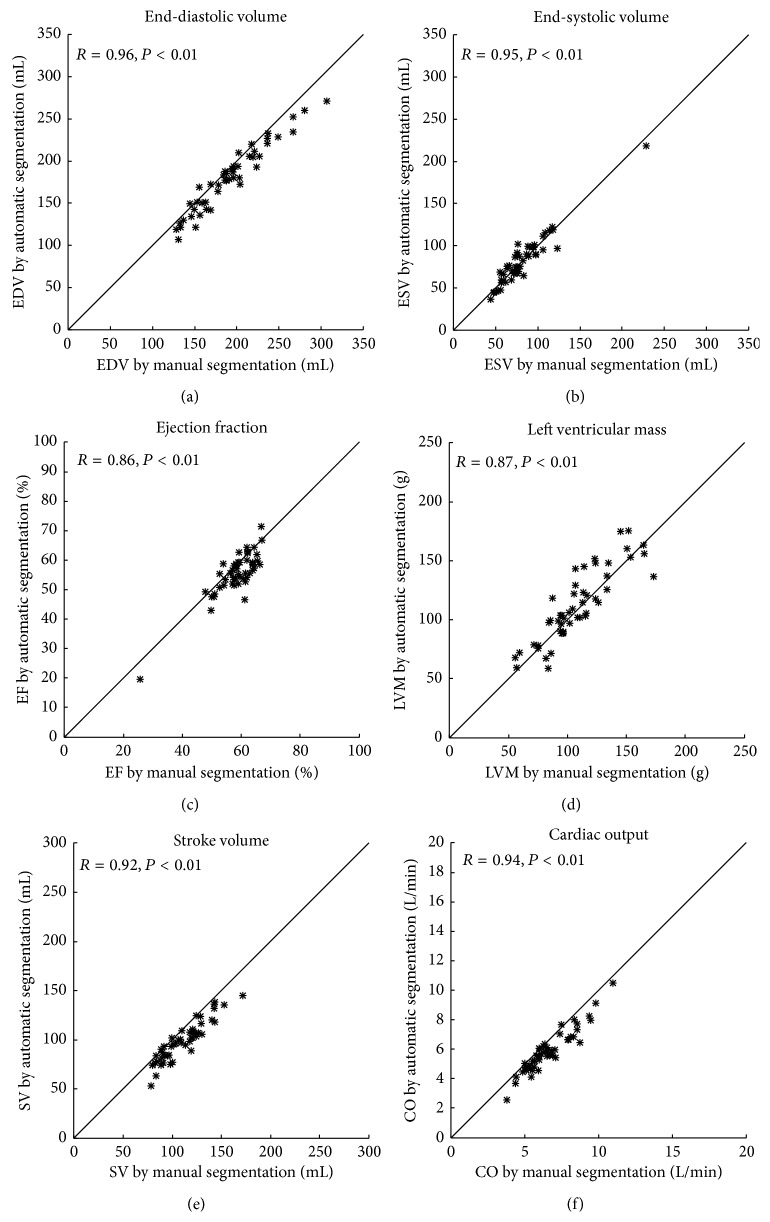
Correlations between automatic segmentation and manual delineation in the test set. Automatic segmentation plotted against manual delineation for end-diastolic volume (EDV, (a)), end-systolic volume (ESV, (b)), ejection fraction (EF, (c)), left ventricular mass (LVM, (d)), stroke volume (SV, (e)) and cardiac output (CO, (f)). The line indicates the line of identity.

**Figure 7:**
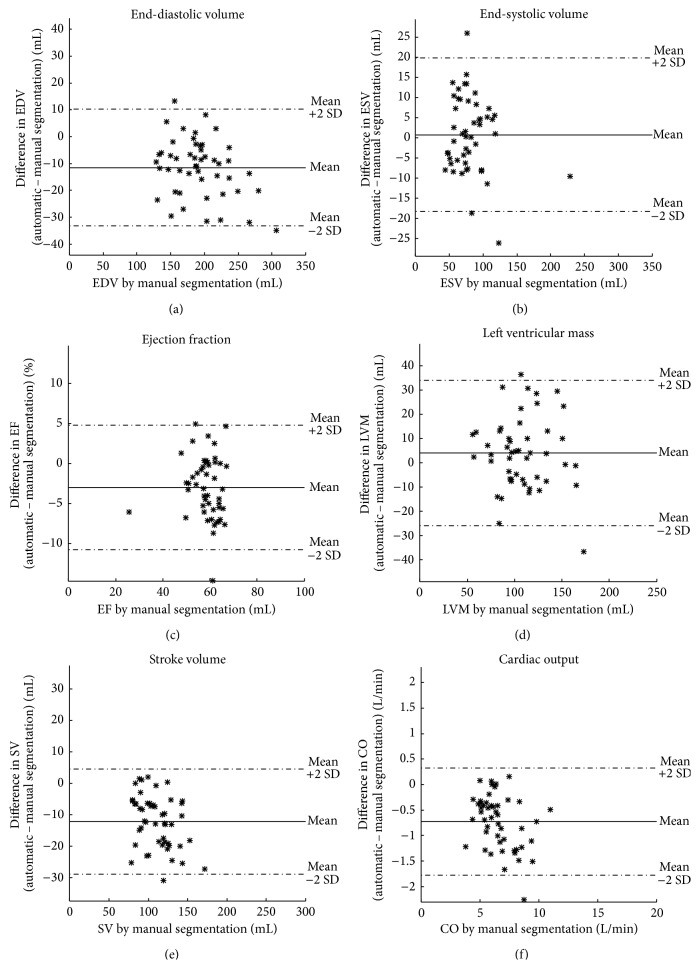
Bias between automatic segmentation and manual delineation in the test set. Differences between automatic segmentation and manual delineation plotted against manual delineation for end-diastolic volume (EDV, (a)), end-systolic volume (ESV, (b)), ejection fraction (EF, (c)), left ventricular mass (LVM, (d)), stroke volume (SV, (e)) and cardiac output (CO, (f)). Solid line indicates mean and dashed lines indicate mean ± 2SD.

Automatic segmentation was performed and compared to manual delineation in the test and compared to interobserver variability in a second observer subset. In one patient the automatic segmentation failed due to a severe bright fold-in artifact connecting the right and left ventricle. This patient was excluded from further analysis resulting in a test set of 49 patients and a second observer subset of 24 patients.  Figure 4 shows an example of automatic segmentation in all slices in end-diastole and end-systole. A comparison between automatic segmentation and manual delineation can be seen in Figure 5 for a basal, midventricular, and apical slice in end-diastole and end-systole. In the additional file a time-resolved 3D-rendering of left ventricle shows the long-axis motion of the epicardial surface resulting from the automatic segmentation algorithm. The differences between automatic segmentation and manual delineation for clinical parameters were EDV −11 ± 11 mL (*R* = 0.96), ESV 1 ± 10 mL (*R* = 0.95), EF −3 ± 4% (*R* = 0.86), LVM 4 ± 15 g (*R* = 0.87), SV −12 ± 8 mL (*R* = 0.92), and CO −0.7 ± 0.5 L/min (*R* = 0.94) (Table 1, Figures 6 and 7). The image processing error measurements were for endocardial segmentation DSC = 0.91 ± 0.03 and P2C = 2.1 ± 0.5 mm and for epicardial segmentation DSC = 0.93 ± 0.02 and P2C = 2.1 ± 0.5 mm as mean over all slices and both end-diastole and end-systole (Table 2). End-diastolic image processing error measurements performed better than end-systolic (Table 2). Midventricular slices performed better than basal and apical slices (Table 3).

In the subset for second observer analysis the differences between second observer manual delineation and reference manual delineation were EDV 10 ± 4 mL, ESV 5 ± 5 mL, EF 0 ± 2%, LVM −7 ± 9 g, SV 5 ± 6 mL, and CO 0.3 ± 0.4 L/min compared to the differences between automatic segmentation and the reference manual delineation which were EDV −9 ± 10 mL, ESV 3 ± 8, EF −3 ± 3%, LVM 2 ± 16 g, SV −12 ± 8 mL, and CO −0.7 ± 0.4 L/min (Table 4). The results for the image processing error measurements DSC and P2C for the second observer subset are given in Tables 5 and 6.

## 4. Discussion 

We have developed an automatic algorithm for time-resolved LV segmentation in magnetic resonance cine balanced steady state free precession (MRSSFP) images. The segmentation is performed in all time frames and all ventricular slices including the slices in which the mitral valve plane and outflow tract move in and out of the slice during a heartbeat. The only manual user input is definition of the most basal and most apical slices including any myocardium in end-diastole. This study brings a state-of-the-art left ventricle segmentation tool to applied clinical research, as the software and source code are provided in open access to researchers. Furthermore, both algorithm and images with ground truth manual delineations are made available for benchmark against future LV segmentation algorithms.

The major algorithmic contributions towards a clinically applicable automatic segmentation method in this study is (1) the use of an EM-algorithm to calculate the distinction between blood, myocardium, and tissues surrounding the heart, (2) removal of papillary muscles by convex hull expansion and expansion to get constant papillary volume, (3) the detection of the outflow tract when moving in and out of the imaging plane, and (4) usage of an optimization step to tune otherwise arbitrary set parameters to the images used.

The algorithm was validated in a test set of 49 subjects and both the clinical parameters, EDV, ESV, EF, and LVM, and the image processing error measurements, DSC and P2C, were reported to allow comparison to errors reported in previous studies. The proposed algorithm has a DSC and P2C error similar to the ones reported in previous studies [3, 4, 12–14]. However, direct comparison between studies is difficult due to differences in methodology. In previous studies it is not defined either how the basal slices were selected, or if the basal slices were excluded or defined separately for end-diastole and end-systole thereby not including the long-axis motion. Furthermore, results may not be directly comparable due to differences in patient population and sequences used for imaging. For instance the test set in the MICCAI challenge [3] was acquired without parallel acquisition techniques which is now clinical standard. In the sequel challenge STACOM [14] not all results were derived using manual delineation as ground truth. A new test set and training set were therefore acquired for this study in order to have images with parallel acquisition, covering all slices and with manual delineation as ground truth. In comparison to the present study, Codella et al. [8, 15] reported better results for all clinical parameters, which is expected with the higher level of user input used in their algorithm LV-METRIC. The present study has a low level of user input with only a selection of slices to include in segmentation. Hu et al. [7] developed a detection of the outflow tract and reported DSC and P2C similar to the present study. However, their method description does not define detection of the outflow tract moving out of the imaging plane. Since the long-axis motion is a major contributor to cardiac pumping [5] it is important to include the basal slices and account for the contraction along the long-axis. Segmentation of the most basal slices with outflow tract becomes more difficult when the myocardium moves in and out of the imaging plane. The proposed algorithm includes all slices with results similar to previous studies not including all basal slices, which brings the algorithm one step closer to automatic LV segmentation applicable for the clinical routine.

The proposed algorithm was compared to interobserver variability of manual delineation in a subset as a major goal of automatic segmentation methods is to reduce observer dependency. The proposed method showed a bias comparable to interobserver variability by manual delineation for the clinical parameters, lower or similar bias for EDV, ESV, and LVM and higher bias for EF, SV, and CO. The SD for the clinical parameters was approximately twice the value found for interobserver variability. The interobserver variability measured as P2C error was overall 1.2 mm compared to 2 mm reported in a previous study [16]. The interobserver variability measured as clinical parameters was overall comparable to those reported in previous studies [17–19]. The standard deviation of LVM for interobserver variability was in this study 7 g which falls within the range of published values from 5 g in a normal material for gradient echo images [20] to 14 g in a study where bSSFP short axis delineations were compared to long-axis delineations [21]. The large range in interobserver variability measurements reported in the literature can most likely be explained by differences in methodology used in the basal regions, differences in image quality, and amount of consensus training. Again many of the studies report differences differently and direct comparisons are difficult.

In order for the algorithm to reach results fully comparable to interobserver variability between two experienced observers, further improvement is needed. By improving the use of the EM-algorithm and by improving the detection of papillary muscles and outflow tract both the accuracy and precision may be reduced. The algorithm might also be further improved to have a smoother segmentation over the cardiac cycle by using more than two time frames in the optimization of parameters and hence possibly get a higher weight on the time dependent parameter. As for all automatic segmentation algorithms a manual approval and possibly manual corrections are needed in a clinical setting.

A limitation to the study is that the training and test set used only patients with coronary artery disease. Other patient categories with, for example, left ventricular dyssynchrony or pronounced trabeculations may need special consideration in the algorithm and further validation.

## 5. Conclusion

We have developed an automatic algorithm for time-resolved segmentation of all LV slices containing any myocardium in magnetic resonance balanced steady state free precession images. The algorithm was quantitatively validated in 49 subjects and both algorithm and images with reference manual delineations are available for benchmark against future LV segmentation algorithms. The algorithm showed a bias comparable to interobserver variability between two experienced observers for the clinical parameters EDV, ESV, EF, LVM, SV, and CO. With a dice and P2C error similar to previous studies the proposed algorithm is favorable due to low level of user input and automatic correction for long-axis motion. The algorithm is one step closer to an automatic segmentation applicable for clinical routine.

## Supplementary Material

The supplementary material shows a movie playing one heart beat in one of the subjects from the test set, with endocardial and epicardial borders by automatic segmentation.

## Figures and Tables

**Table 1:** Clinical parameters in test set. Results for clinical parameters in the full test set (*n* = 49) as differences between automatic segmentation and manual delineation.

	Absolute difference	Relative difference	*P* value
EDV	−11 ± 11 mL	−6 ± 6%	<0.01
ESV	1 ± 10 mL	1 ± 13%	0.57
EF	−3 ± 4%	−5 ± 7%	<0.01
LVM	4 ± 15 g	4 ± 14%	0.07
SV	−12 ± 8 mL	−11 ± 8%	<0.01
CO	−0.7 ± 0.5 L/min	−11 ± 8%	<0.01

Absolute and relative values are expressed as mean ± SD. EDV = end-diastolic volume, ESV = end-systolic volume, EF = ejection fraction, and LVM = left ventricular mass.

**Table 2:** Image processing error measurement in test set. Image processing error measurements in the full test set (*n* = 49) as dice similarity coefficient (DSC) and point to curve (P2C) between automatic segmentation and manual delineation.

	Dice similarity coefficient (DSC)	Point to curve (P2C)
Endocardium overall	0.91 ± 0.03	2.1 ± 0.5 mm
Endocardium ED	0.93 ± 0.03	1.9 ± 0.6 mm
Endocardium ES	0.85 ± 0.04	2.3 ± 0.5 mm
Epicardium overall	0.93 ± 0.02	2.1 ± 0.5 mm
Epicardium ED	0.94 ± 0.02	2.1 ± 0.6 mm
Epicardium ES	0.91 ± 0.03	2.2 ± 0.7 mm

Differences are expressed as mean ± SD. For a perfect overlap between the regions DSC should be 1 and P2C should be 0. ED = end diastole, ES = end systole.

**Table 3:** Image processing error measurements in test set divided into slice sections. Image processing error measurements in the full test set (*n* = 49) as dice similarity coefficient (DSC) and point to curve (P2C) between automatic segmentation and manual delineation.

	Dice similarity coefficient (DSC)	Point to curve (P2C)
Endocardium basal	0.88 ± 0.06	2.7 ± 1.0 mm
Endocardium midventricular	0.94 ± 0.02	1.6 ± 0.4 mm
Endocardium apical	0.89 ± 0.03	2.1 ± 0.7 mm
Epicardium basal	0.89 ± 0.05	3.3 ± 1.2 mm
Epicardium midventricular	0.96 ± 0.02	1.3 ± 0.5 mm
Epicardium apical	0.92 ± 0.03	2.2 ± 0.8 mm

Differences are expressed as mean ± SD. For a perfect overlap between the regions DSC should be 1 and P2C should be 0. Basal, midventricular, and apical sections are defined as 1/3 each of the ventricular length in end diastole and end systole separately.

**Table 4:** Clinical parameters in second observer subset. Differences for clinical parameters in the second observer subset (*n* = 24) for second observer manual delineation versus manual reference delineation and for automatic segmentation versus manual reference delineation.

	Automatic segmentation versus manual reference			Second observer versus manual reference		
	Absolute difference	Relative difference	*P* value	Absolute difference	Relative difference	*P* value
EDV	−9 ± 10 mL	−5 ± 5%	<0.01	10 ± 4 mL	6 ± 2%	<0.01
ESV	3 ± 8 mL	4 ± 12%	0.1	5 ± 5 mL	6 ± 6%	<0.01
EF	−3 ± 3%	−6 ± 6%	<0.01	0 ± 2%	−1 ± 4%	0.44
LVM	2 ± 16 g	3 ± 13%	0.55	−7 ± 9 g	−7 ± 8%	<0.01
SV	−12 ± 8 mL	−10 ± 6%	<0.01	5 ± 6 mL	5 ± 5%	<0.01
CO	−0.7 ± 0.4 L/min	−10 ± 6%	<0.01	0.3 ± 0.4 L/min	5 ± 5%	<0.01

Absolute and relative difference expressed as mean ± SD. EDV = end-diastolic volume, ESV = end-systolic volume, EF = ejection fraction, and LVM = left ventricular mass.

**Table 5:** Image processing error measurements in second observer subset. Image processing error measurements in the second observer subset (*n* = 24) as dice similarity coefficient (DSC) and point to curve (P2C) for second observer manual delineation versus manual reference delineation and for automatic segmentation versus manual reference delineation.

	Automatic segmentation versus manual reference		Second observer versus manual reference	
	DSC	P2C	DSC	P2C
Endocardium overall	0.91 ± 0.02	2.0 ± 0.4 mm	0.95 ± 0.01	1.2 ± 0.2 mm
Endocardium ED	0.93 ± 0.02	1.8 ± 0.5 mm	0.96 ± 0.01	1.1 ± 0.3 mm
Endocardium ES	0.85 ± 0.04	2.4 ± 0.5 mm	0.92 ± 0.03	1.4 ± 0.3 mm
Epicardium overall	0.93 ± 0.01	2.2 ± 0.4 mm	0.96 ± 0.01	1.2 ± 0.33 mm
Epicardium ED	0.94 ± 0.01	2.0 ± 0.5 mm	0.97 ± 0.01	1.1 ± 0.4 mm
Epicardium ES	0.91 ± 0.02	2.4 ± 0.6 mm	0.95 ± 0.01	1.4 ± 0.4 mm

Difference are expressed as mean ± SD. For a perfect overlap between the regions DSC should be 1 and P2C should be 0. ED = end diastole, ES = end systole.

**Table 6:** Image processing error measurements in second observer set divided into slice sections. Image processing error measurements in the second observer subset (*n* = 24) as dice similarity coefficient (DSC) and point to curve (P2C) for second observer manual delineation versus manual reference delineation and for automatic segmentation versus manual reference delineation.

	Automatic segmentation versus manual reference		Second observer versus manual reference	
	DSC	P2C	DSC	P2C
Endocardium basal	0.88 ± 0.06	2.7 ± 1.1 mm	0.94 ± 0.02	1.5 ± 0.4 mm
Endocardium midventricular	0.94 ± 0.01	1.6 ± 0.4 mm	0.96 ± 0.01	1.1 ± 0.3 mm
Endocardium apical	0.90 ± 0.03	2.0 ± 0.7 mm	0.94 ± 0.01	1.1 ± 0.3 mm
Epicardium basal	0.89 ± 0.05	3.3 ± 1.2 mm	0.95 ± 0.02	1.5 ± 0.6 mm
Epicardium midventricular	0.96 ± 0.01	1.3 ± 0.4 mm	0.97 ± 0.01	0.8 ± 0.2 mm
Epicardium apical	0.92 ± 0.02	2.3 ± 0.7 mm	0.95 ± 0.01	1.4 ± 0.5 mm

Difference are expressed as mean ± SD. For a perfect overlap between the regions DSC should be 1 and P2C should be 0. Basal, midventricular, and apical sections are defined as 1/3 each of the ventricular length in end diastole and end systole separately.
